# Suicidal Thoughts and Behaviors Among Chinese Adolescents in Relation to Negative Life Events, Internet Addiction, and Sexual Abuse: Cross-Sectional Study

**DOI:** 10.2196/85371

**Published:** 2026-03-25

**Authors:** Juanfang Zhu, Qinqin Jiang, Feng Zhang, Hao Liu, Shaojie Yu, Jinhai Sun, Zhe Zhao, Lijuan Liu, Lei Yuan

**Affiliations:** 1 Faculty of Military Health Service, Naval Medical University, No. 800 Xiangyin Road, Shanghai, 200433, China, 86 021-81871450, 86 021-81871404; 2 College of Health Management, Southern Medical University, Guangzhou, China

**Keywords:** suicidal thoughts, suicidal behaviors, sexual abuse, SA, internet addiction, IA, chain mediation effect, adolescents

## Abstract

**Background:**

Increasing suicidal thoughts and behaviors (STB) among adolescents raise social concerns and have a well-recognized association with sexual abuse (SA). However, research regarding the mechanisms explaining the association between SA and STB remains limited.

**Objective:**

This study aims to examine the chained mediating effects of negative life events (NLE) and internet addiction (IA) between SA and STB among adolescents in China.

**Methods:**

This cross-sectional study used data from the Science Database of the People Mental Health survey conducted between March 2013 and December 2022 by the National Population Health Data Center of the National Research Institute for Family Planning. Through stratified sampling, 20,893 adolescents were recruited from 16 Chinese provinces. After excluding samples with missing relevant variables, 10,664 (55.89%; aged 16-17.9 y; n=5826, 54.63% women) adolescents were included in the final analysis. STB was the outcome variable, with NLE and IA as mediators, all assessed via a questionnaire that was uniformly administered by trained investigators in school settings. The Pearson χ^2^ test was used to analyze the association between SA and STB. Using a combination of multiple linear regression and bootstrap testing, the study constructed a chain mediation model to explore how SA influences STB in adolescents through NLE and IA.

**Results:**

The scores for SA, NLE, IA, and STB were 1.330 (SD 1.714), 51.960 (SD 23.822), 34.88 (SD 13.852), and 0.690 (SD 1.396), respectively. Multiple linear regression analysis indicated SA was associated with NLE (β=2.382, 95% CI 2.112‐2.653; *P*<.001), IA (β=1.094, 95% CI 0.939‐1.249; *P*<.001), and STB (β=0.135, 95% CI 0.119‐0.151; *P*<.001). Mediation analysis revealed a significant direct association between SA and STB (74.81%; β=0.101, 95% CI 0.085‐0.117; *P*<.001). Indirect effects constituted 25.19% of the total association (β=0.034, 95% CI 0.029‐0.040; *P*<.001), comprised of 3 specific pathways: via NLE (11.85%; β=0.016, 95% CI 0.012‐0.020; *P*<.001), via IA (10.37%; β=0.014, 95% CI 0.010‐0.018; *P*<.001), and via the sequential pathway through NLE and then IA (2.96%; β=0.004, 95% CI 0.003‐0.006; *P*<.001).

**Conclusions:**

Unlike previous research that examined them separately, this study reveals the chain mediation mechanism of NLE and IA in the relationship between SA and STB, providing a new research perspective on the complex evolution from early adversity to extreme outcomes. This research also provided clear direction and scientific evidence for the development of comprehensive suicide prevention strategies. Future efforts should focus on the prevention of SA and trauma intervention while simultaneously strengthening adolescents’ cognitive reappraisal of negative events and guiding their online behavior.

## Introduction

Adolescents’ susceptibility to suicidal thoughts and behaviors (STB) raises social and academic concerns [1-3]. According to the World Health Organization (WHO) estimates, suicide was the fourth leading cause of mortality for youths aged 15 to 29 years globally in 2019 [4], with significant variations observed across countries, age groups, and genders [5]. Both attempted and completed suicides impose substantial social, economic, and emotional burdens worldwide. Despite efforts to reduce suicide risk, global rates of youth STB exhibit no improvement [6]. Hence, the identification of risk factors and antecedents predisposing individuals to increased suicide risk is imperative to enable prompt and appropriate monitoring and intervention [7]. Sexual abuse (SA) is a recognized risk factor for suicidal ideation and behavior [8,9]. By disrupting normal neuroendocrine and immune functions [10], SA exerts a direct effect on increasing suicide risk [11]. Accordingly, this study proposes hypothesis 1 as follows: SA is positively associated with STB in adolescents. Although this association is well-established, the mediating mechanisms through which SA elevates suicide risk remain unclear, particularly among Chinese adolescents.

Based on theoretical reasoning, negative life events (NLE) and internet addiction (IA) may serve as potential mediators. According to the integrated motivational-volitional model of STB, NLE are the main stressors driving STB, which play a “trigger” role [12] and are strong predictors thereof [13]. Previous cross-sectional studies have linked NLE such as academic pressure and interpersonal conflict to suicidal ideation and behavior in university students [14,15], an association also supported by longitudinal research [16]. Furthermore, evidence suggests that survivors of SA are more likely to experience other forms of interpersonal harm, including physical assault [17], illicit drug dependence [18], and adolescent dating violence [19]—all constituting NLE. Thus, hypothesis 2 is proposed as follows: NLE mediate the relationship between SA and STB. Recent studies indicate a significant association between adolescent IA and suicidal ideation or behavior [17,20,21], with addicted adolescents exhibiting higher suicide risk. A key risk factor for IA may be childhood trauma, which is strongly predictive of lifelong mental disorders [22,23]. A history of childhood trauma appears equally prevalent among individuals with IA and those with substance dependence [24]. Moreover, a study of Chinese university students found that childhood trauma significantly influences suicidal ideation, an effect mediated by IA [25]. Hence, hypothesis 3 is proposed as follows: IA mediates the relationship between SA and STB. Due to underdeveloped social-cognitive abilities and the inevitability of academic and interpersonal stressors, adolescents often experience NLE. While the causes of IA are still under investigation, NLE are considered a key contributing factor. Research shows a significant positive correlation between NLEs and IA among university students [26-29], a link observed across subtypes such as interpersonal distress, academic pressure, punishment, and health-related problems [30]. Consequently, hypothesis 4 is proposed as follows: NLEs and IA operate as serial mediators in the relationship between SA and STB.

While existing research has separately revealed significant associations between SA and STB, between NLEs and STB, and between IA and STB, how these factors interact within a coherent mechanistic model to collectively explain the pathway from early trauma to ultimate risk remains poorly understood [8-11,14,15,17,21,26]. Building on prior findings, this study aims to use a large representative sample of youth to examine the association between SA and STB, and to explore the potential direct and indirect effects of SA on STB mediated through NLE and IA. This investigation seeks to deepen the understanding of the mechanisms underlying adolescent suicide risk and to provide an evidence-based roadmap for the precise prevention and tiered intervention of suicide risk among Chinese adolescents.

## Methods

### Data and Sampling

**Figure 1.**
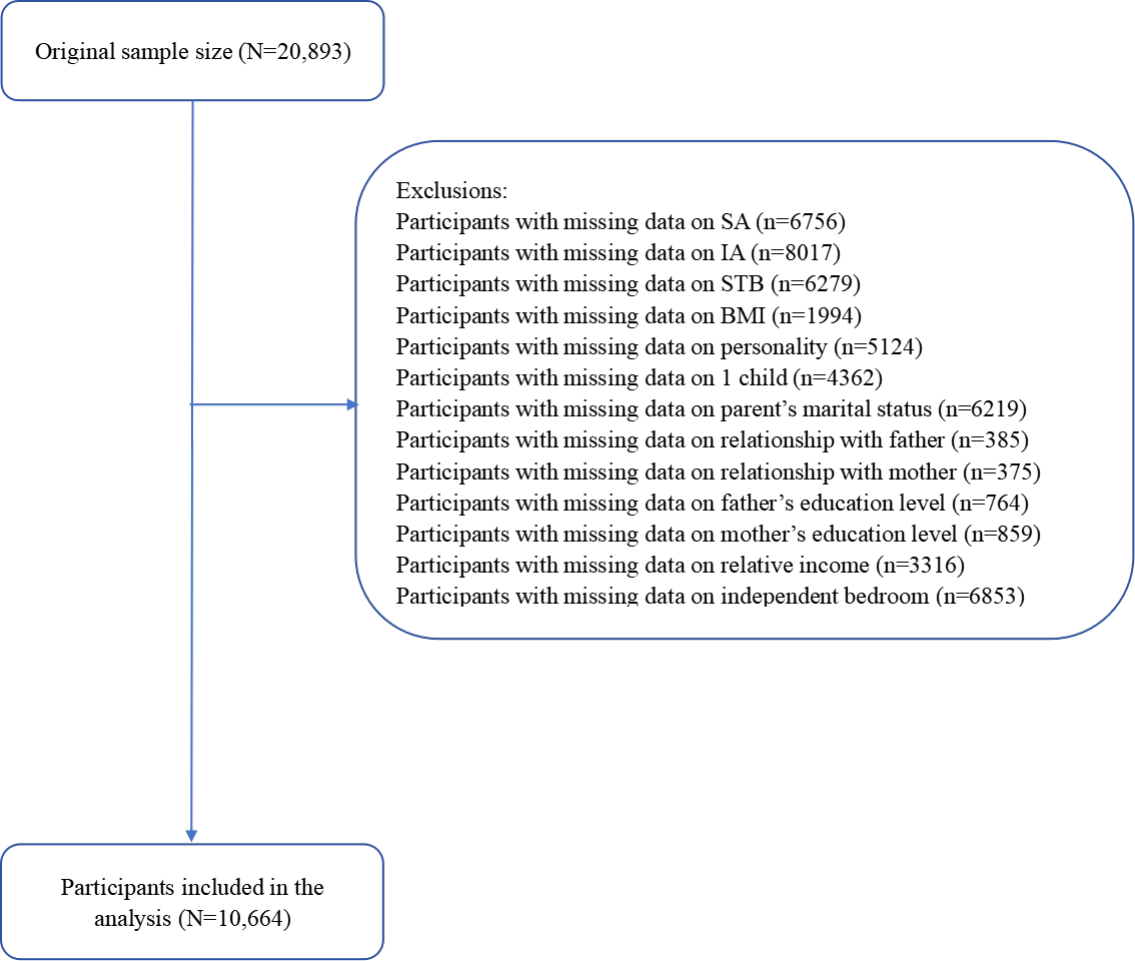
Flowchart of adolescent participants in the cross-sectional study on sexual abuse (SA) and suicidal thoughts and behaviors (STB), China, 2013‐2022. IA: internet addiction

This study used data from the Science Database of the People Mental Health survey conducted between March 2013 and December 2022 by the National Population Health Data Center of the National Research Institute for Family Planning [31]. The data are designed horizontally and vertically, and through dynamic, continuous, and systematic collection of mental health and related risk factor information from children, adolescent, young adult, middle-aged, and older adult populations, as well as from specific populations, occupational populations, and mental health specialties. Thereby, the trajectory of changes in the mental health of individuals, clusters, and populations is presented, and the knowledge mapping of mental health data is developed. The surveys adhere to psychological industry standards, expert consensus, guidelines, and regulations, using widely accepted international and domestically standardized scales adapted for Chinese norms. Data collection involved validity and reliability assessments and was conducted using standardized procedures for both offline and online administration, using surveys and assessments within a mental health context. A multistage stratified random sampling method was used in this study. From March 2013 to December 2022, 20,893 adolescents were recruited from secondary schools across 16 provinces in China. For this analysis, after excluding respondents with missing values on any variable, the final analytical sample comprised 10,664 adolescents. Figure 1 illustrates the data cleaning process. Prior to the survey, informed consent was obtained from both the respondents’ parents and the respondents themselves, following local parental consent procedures.

### Measures

#### Sociodemographic Variables

Sociodemographic variables included age, gender, ethnic group, BMI, personality type, 1-child status, parents’ marital status, relationship with father, relationship with mother, father’s educational level, mother’s educational level, region, relative income, and having an independent bedroom. Regions, categorized according to the affiliation of the respondent’s province in the *Chinese Health Statistics Yearbook* [32], were divided into central, western, and eastern regions.

#### Grouping Variable

This study’s grouping variable is hukou, which is categorized into urban and rural areas.

#### Suicidal Thoughts and Behaviors

Suicidal behaviors are measured by using the following 3 items, according to the Global School-Based Student Health Survey, including suicide ideation, plans, and attempts, respectively: “Have you seriously considered suicide in the last 12 months?” “Have you made a specific plan to commit suicide in the last 12 months?” and “Have you attempted suicide in the last 12 months?” All 3 variables were considered dummy variables, with “never,” “≤2 days,” and “>2 days” coded as 0, 1, and 2, respectively. The total score for STB ranges from 0 to 6, with a higher score indicating more STB. In this study sample, the Cronbach α coefficient for the STB assessment was 0.713, the Kaiser-Meyer-Olkin (KMO) value was 0.667, and the Bartlett test of sphericity yielded a significant result (*P*<.001).

#### Sexual Abuse

Respondents were asked the following 9 questions to measure SA: “Has anyone ever kissed you without your consent?” “Has anyone ever exposed their genitals to you?” “Has anyone ever masturbated in front of you?” “Has anyone ever told you dirty jokes, made pornographic gestures, or shown you pornographic pictures?” “Has anyone ever touched your sensitive parts (eg, lower body, breasts, and genitals)?” “Has anyone ever rubbed their genitals on you?” “Has anyone ever made oral contact with your sex organ?” “Has anyone ever tried to have sex with you?” and “Has anyone ever forced sex on you?” All 9 variables were considered dummy variables, with “yes” and “no” coded as 1 and 0, respectively. In this study sample, the Cronbach α coefficient for the SA experience assessment was 0.747, the KMO value was 0.850, and the Bartlett test of sphericity yielded a significant result (*P*<.001).

#### Negative Life Events

This study measured NLE using the ASLEC (Adolescent Self-Rating Life Event Checklist; Checklist 2) [33], which is used to assess adverse events within the past 12 months. The ASLEC comprises 27 items in the following 6 dimensions: interpersonal relationships (interpersonal conflict or contradiction), study pressure (stress caused by learning), being punished (punishment by caregivers, teachers, or peers), bereavement (death of beloved ones), health adaptation, and other dimensions (eg, school refusal) [33]. The ASLEC assesses the frequency and intensity of life events experienced by respondents in the past half-year. Respondents rate each NLE’s impact on a 5-point Likert scale (1=never to 5=extremely severe). The ASLEC’s total score ranges between 27 and 135, with a higher score indicating a greater number of NLE and greater stress experienced over the past half-year. Previous studies have reported that this scale is reliable and valid among Chinese children [27,34]. In this study sample, the Cronbach α coefficient for the Adolescent Life Events Scale was 0.965, the KMO value was 0.971, and the Bartlett test of sphericity yielded a significant result (*P*<.001).

#### Internet Addiction

The research focused predominantly on IA as measured by the Internet Addiction Test (IAT) developed by Young [35]. The IAT is a 20-item questionnaire that measures characteristics and behaviors associated with compulsive internet use. Each item is weighted using a Likert scale ranging from 1 (“rarely”) to 5 (“always”). The IAT scale comprises the following 6 subscales: salience, excessive use, neglect of work, anticipation, lack of control, and neglect of social life; however, precise interval thresholds are not defined for this scale. Therefore, a higher score represents greater addiction. Previous studies have reported that this scale is reliable and valid among Chinese children [36,37]. In this sample, the scale demonstrated excellent internal consistency, with a Cronbach α coefficient of 0.947. KMO measure of sampling adequacy was 0.959, and the Bartlett test of sphericity was significant (*P*<.001).

**Figure 2.**
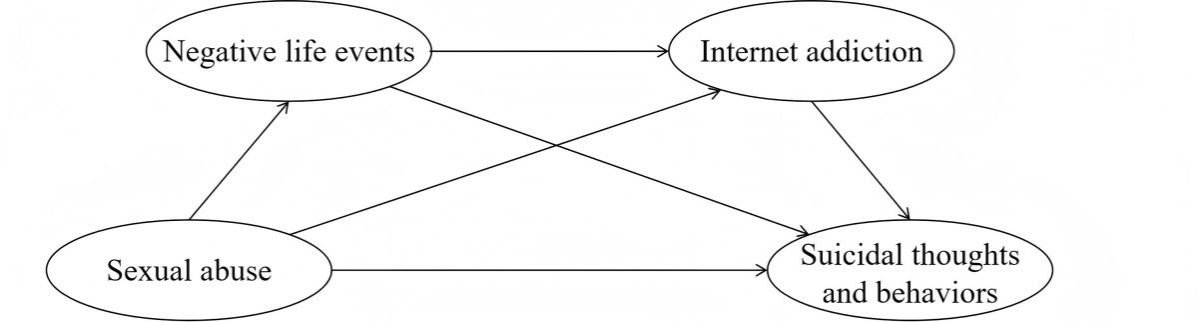
Proposed serial mediation model depicting the relationships among sexual abuse, negative life events, internet addiction, and suicidal thoughts and behaviors in Chinese adolescents.

Figure 2 illustrates the proposed serial mediation model.

### Statistical Analysis

Data analysis was performed using Stata (version 18.0; StataCorp LLC) and SPSS (version 26.0; IBM Corp) software. The study was reported following the Reporting Standards for Studies Using Structural Equation Modeling to ensure compliance with the transparency and completeness requirements stipulated by the JARS (Journal Article Reporting Standards) guidelines (Checklist 1) [38,39]. Descriptive statistics were computed for respondents’ sociodemographic characteristics. Frequencies and percentages were reported for categorical variables, whereas means and SDs were reported for continuous variables. Associations between independent and outcome variables were estimated using the Pearson chi-square test (or 2-tailed *t* test for continuous variables). The Pearson correlation analysis measures the strength and direction of a linear relationship between dependent and independent variables. Using a combination of multiple linear regression and bootstrap testing, we constructed a chain mediation model. Multivariate linear regression analysis was used to identify significant independent correlates of STB. To test the hypothesized mediation effects, we conducted path analysis using the bootstrap method, with 5000 bootstrap resamples to generate bias-corrected 95% CI. This approach quantified the indirect effects of the independent variable on the dependent variable through the mediators, as well as the direct effects after controlling for the mediators. The 2-tailed significance level was set at *P*<.05.

### Ethical Considerations

This study is a secondary analysis based on the Science Database of the People Mental Health Survey. The original data collection adhered to the principles of the Declaration of Helsinki, and written informed consent was obtained from all participants and their guardians. This research used only processed, publicly available data containing no personally identifiable information and involved no interaction with human participants. No identification of individual participants is presented in any images within this study or supplementary materials. The Science Database of the People Mental Health Survey has specific licensing and citation requirements. The authors obtained permission to use the data and have strictly complied with the relevant data use agreement regarding noncommercial academic research and proper citation. This study was exempted from additional ethics review and approval by the Naval Medical University Ethics Committee.

## Results

### Sociodemographic Characteristics

Among the participants, 2075 (19.46%), 5960 (55.89%), and 2629 (24.65%) were aged younger than 16 years, 16 to 17.9 years, and 18 years or older, respectively; 4838 (45.37%) and 5826 (54.63%) were male and female adolescents, respectively; 5703 (53.48%) and 4961 (46.52%) were Han Chinese and belonged to an ethnic minority, respectively; 6796 (63.73%), 2511 (23.55%), 998 (9.36%), and 359 (3.37%) had BMIs in the ranges of 18.5 to 23.9 kg/m^2^, <18.5 kg/m^2^, 24 to 27.9 kg/m^2^, and ≥28 kg/m^2^, respectively; 1430 (13.41%), 3355 (31.46%), and 5879 (55.13%) were introverts, extroverts, and ambiverts, respectively; 3099 (29.06%) had 1-child status in their families; 421 (3.95%) and 369 (3.46%) had divorced parents and remarried families, respectively; 8833 (82.83%) and 9712 (91.07%) had amicable relationships with their fathers and mothers, respectively; 1012 (9.49%) and 915 (8.58%) respondents’ fathers and mothers had an educational level of college and higher, respectively; 4898 (45.93%), 2265 (21.24%), and 3501 (32.83%) were in the central, eastern, and western regions, respectively; 6247 (58.58%), 2539 (23.81%), and 1878 (17.61%) had general, rich, and poor income statuses, respectively; and 8910 (83.55%) had an independent bedroom. The chi-square test and 2-tailed *t* test revealed differences in age, gender, ethnic group, BMI, 1-child status, parents’ marital status, father’s educational level, mother’s educational level, relative income, and independent bedroom of middle school adolescents between urban and rural areas (Table 1). Gender differences exist in the prevalence of SA (57.24%) and STB (24.8%) among adolescent samples in China currently (Table S1 in Multimedia Appendix 1).

**Table 1.** Characteristics of Chinese adolescent participants (N=10,664).

Variables	Total (N=10,664)	Rural (n=8028)	Urban (n=2636)	Statistics, chi-square (*df*)	*P* value
Age (y), n (%)				9.3 (2)	.01
<16	2075 (19.46)	1521 (18.95)	554 (21.02)		
16-17.9	5960 (55.89)	4479 (55.79)	1481 (56.18)		
≥18	2629 (24.65)	2028 (25.26)	601 (22.80)		
Gender, n (%)				30.3 (1)	<.001
Woman	5826 (54.63)	4508 (56.15)	1318 (50)		
Man	4838 (45.37)	3520 (43.85)	1318 (50)		
Ethnic group, n (%)				30.3 (1)	<.001
Han Chinese	5703 (53.48)	4171 (51.96)	1532 (58.12)		
Ethnic minority	4961 (46.52)	3857 (48.04)	1104 (41.88)		
BMI (kg/m^2^), n (%)				34.0 (3)	<.001
18.5~23.9	6796 (63.73)	5222 (65.05)	1574 (59.71)		
<18.5	2511 (23.55)	1853 (23.08)	658 (24.96)		
24.0~27.9	998 (9.36)	714 (8.89)	284 (10.77)		
≥28.0	359 (3.37)	239 (2.98)	120 (4.55)		
Personality, n (%)				13.3 (2)	<.001
Introvert	1430 (13.41)	1107 (13.79)	323 (12.25)		
Extrovert	3355 (31.46)	2454 (30.57)	901 (34.18)		
Ambivert	5879 (55.13)	4467 (55.64)	1312 (53.57)		
One child, n (%)				1200.0 (1)	<.001
No	7565 (70.94)	6397 (79.68)	1168 (44.31)		
Yes	3099 (29.06)	1631 (20.32)	1468 (55.69)		
Parent’s marital status, n (%)				66.7 (3)	<.001
Married	9412 (88.26)	7161 (89.02)	2251 (85.39)		
Divorce	421 (3.95)	275 (3.43)	146 (5.54)		
Remarry	369 (3.46)	227 (2.83)	142 (5.39)		
Other	462 (4.33)	365 (4.55)	97 (3.68)		
Relationship with father, n (%)				7.5 (2)	.02
Well	8833 (82.83)	6671 (83.10)	2162 (82.02)		
General	1449 (13.59)	1092 (13.06)	357 (13.54)		
Other	382 (3.58)	265 (3.30)	117 (4.44)		
Relationship with mother, n (%)				12.2 (2)	.34
Well	9712 (91.07)	7302 (90.96)	2410 (91.43)		
General	768 (7.20)	579 (7.21)	189 (7.17)		
Other	184 (1.73)	147 (1.83)	37 (1.40)		
Father’s education level, n (%)				1900.0 (3)	<.001
Primary school and below	2341 (21.95)	2142 (26.68)	199 (7.55)		
Junior high school	4993 (46.82)	4154 (51.74)	839 (31.83)		
High school	2318 (21.74)	1449 (18.05)	869 (32.97)		
College and higher	1012 (9.49)	283 (3.53)	729 (27.66)		
Mother’s education level, n (%)				2200.0 (3)	<.001
Primary school and below	3392 (31.8)	3103 (38.65)	289 (10.96)		
Junior high school	4558 (42.74%)	3709 (46.20)	849 (32.21)		
High school	1799 (16.87)	994 (12.38)	805 (30.54)		
College and higher	915 (8.58)	222 (2.77)	693 (26.29)		
Region				111.8 (2)	<.001
Central region	4898(45.93%)	3920(48.83%)	978(37.10%)		
Eastern region	2265(21.24%)	1636(20.38%)	629(23.86%)		
Western region	3501(32.83%)	2472(30.79%)	1029(39.04%)		
Relative income, n (%)				550.0(2)	<.001
General	6247 (58.58)	4843 (60.33)	1404 (53.26)		
Rich	2539 (23.81)	1511 (18.82)	1028 (39)		
Poor	1878 (17.61)	1674 (20.8)	204 (7.74)		
Independent bedroom, n (%)				35.6 (1)	<.001
No	1754 (16.45)	1419 (17.68)	335 (12.71)		
Yes	8910 (83.55)	6609 (82.32)	2301 (87.29)		
Sexual abuse, mean (SD)	—^a^	1.289 (1.672)	1.436 (1.832)	−3.289^b^	.001
Negative life events, mean (SD)	—	52.734 (23.953)	49.596 (23.266)	5.876^c^	<.001
Internet addiction, mean (SD)	—	34.628 (13.777)	35.650 (14.056)	−3.290^c^	.001
Suicidal thoughts and behaviors, mean (SD)	—	0.648 (1.370)	0.805 (1.469)	−5.010^c^	<.001

a Not applicable.

b Rank-sum test.

c 2-tailed* t* test.

### Correlation Analyses

Table 2 presents the means, SD, and Pearson correlations of all study variables. Notably, SA was significantly positively correlated with NLE (*r*=0.174; *P*<.001), IA (*r*=0.199; *P*<.001), and STB (*r*=0.137; *P*<.001). Moreover, NLE were significantly positively correlated with IA (*r*=0.281; *P*<.001) and STB (*r*=0.173; *P*<.001). Finally, IA was significantly positively correlated with STB (*r*=0.197; *P*<.001). Significant correlations between the variables initially supported our hypotheses.

**Table 2.** Means, SDs, and correlation of sex abuse, negative life events, internet addiction, and suicidal thoughts and behaviors (STB; N=10,664).

Variable	Value, mean (SD)	1^a^	2^b^	3^c^	4^d^
Sexual abuse	1.330 (1.714)	1.000			
Negative life events	51.960 (23.822)	0.174^e^	1.000		
Internet addiction	34.88 (13.852)	0.199^e^	0.281^e^	1.000	
STB	0.690 (1.396)	0.137^e^	0.173^e^	0.197^e^	1.000

a Correlations between sexual abuse and itself, negative life events, internet addiction, and STB.

b Correlations between negative life events and itself, internet addiction, and STB.

c Correlations between internet addiction and itself and STB.

d Correlation of STB with itself.

e *P*<.001.

### Chain Mediation Analysis

**Figure 3.**
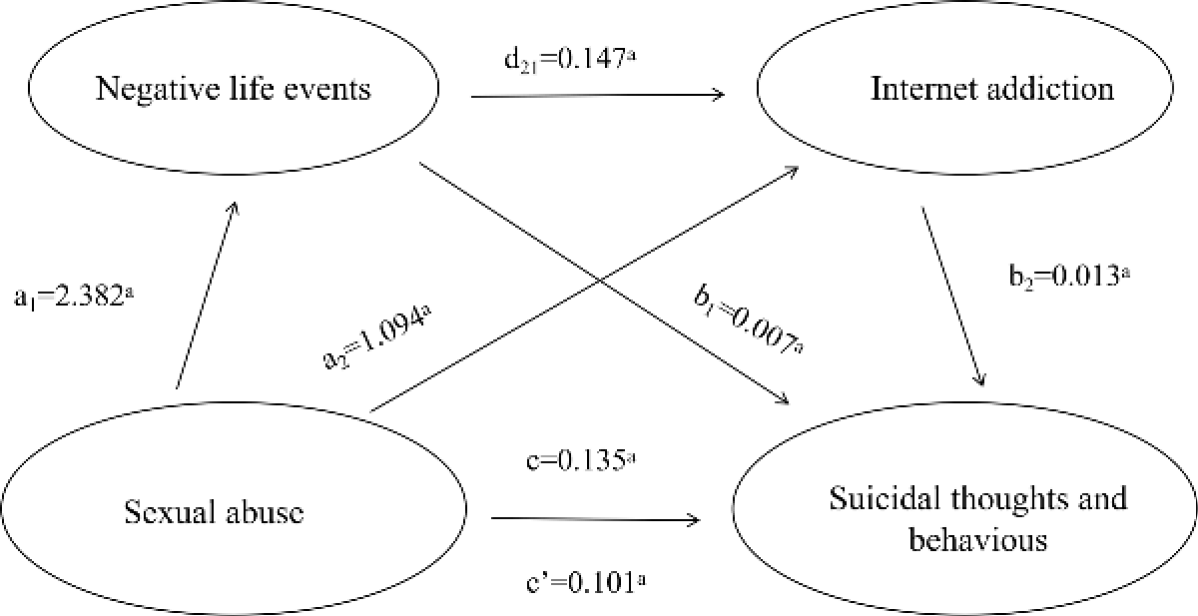
The indirect effects of negative life events and internet addiction in the relationship between sexual abuse and suicidal thoughts and behaviors. a: *P*<.001.

To control for the influence of potential confounding factors, covariates were included in all mediation effect test models. We constructed a chain mediation effect model based on different theoretical assumptions (Figure 3). Multicollinearity tests indicated that the variance inflation factor values for all predictors were well below the critical threshold of 5, suggesting no serious multicollinearity issues in the model (Table S2 in Multimedia Appendix 1).

Figure 3 depicts the tested structural model, which statistically demonstrated significant associations among key variables. The model indicated that a higher level of SA was associated with higher levels of NLE (β=2.382, 95% CI 2.112‐2.653; *P*<.001), IA (β=1.094, 95% CI 0.939‐1.249; *P*<.001), and STB (β=0.135, 95% CI 0.119‐0.151; *P*<.001). Higher levels of NLE were further associated with higher levels of IA (β=0.147, 95% CI 0.136‐0.158; *P*<.001) and STB (β=0.007, 95% CI 0.005‐0.008; *P*<.001). IA also showed a significant association with higher levels of STB (β=0.013, 95% CI 0.011‐0.015; *P*<.001). Furthermore, as presented in Table 3, the direct effect of SA on STB was significant (β=0.101, 95% CI 0.085‐0.117; *P*<.001), accounting for 74.81 % of the total effect. The indirect effect of SA on STB through NLE was significant (β=0.016, 95% CI 0.012‐0.020; *P*<.001), accounting for 11.85% of the total effect; the indirect effect of SA on STB through IA was significant (β=0.014, 95% CI 0.010‐0.018; *P*<.001), accounting for 10.37% of the total effect. Moreover, the indirect effect of SA on STB through NLE and IA was significant (β=0.004, 95% CI 0.003‐0.006; *P*<.001), accounting for 2.96% of the total effect. All regression coefficients are unstandardized coefficients.

**Table 3.** Results of the chain mediation model analyzing the pathway from sexual abuse to suicidal thoughts and behaviors via negative life events and internet addiction.

Model pathways	β^a^ (95% CI)		*P* value	Ratio (%)
Total effect	0.135 (0.119-0.151)		<.001	100.00
Direct effect	0.101 (0.085-0.117)		<.001	74.81
Total indirect effect	0.034 (0.029-0.040)		<.001	25.19
Sexual abuse →negative life events→ suicidal thoughts and behaviors	0.016 (0.012-0.020)		<.001	11.85
Sexual abuse → Internet addiction→ suicidal thoughts and behaviors	0.014 (0.010-0.018)		<.001	10.37
Sexual abuse →negative life events→ internet addiction →suicidal thoughts and behaviors	0.004 (0.003-0.006)		<.001	2.96

a Unstandardized regression coefficient.

### Missing Data

Among the 18 variables analyzed in this study, the variables NLE, Age, gender, ethnic group, and region have no missing values. The proportion of missing data is presented in Table 4. The Little test for completely random missingness showed a significant result (*χ*²_8_=14670.975; *P*<.001).

**Table 4.** The proportion of missing data.

Variables	Missing value, n (%)	
SA^a^	6756 (32.3)	
NLE^b^	0 (0)	
IA^c^	8017 (38.4)	
STB^d^	6279 (30.1)	
Age	0 (0)	
Gender	0 (0)	
Ethnic group	0 (0)	
BMI	1994 (9.5)	
Personality	5124 (24.5)	
One child	4362 (20.9)	
Parent’s marital status	6219 (29.8)	
Relationship with father	385 (1.8)	
Relationship with mother	375 (1.8)	
Father’s education level	764 (3.7)	
Mother’s education level	859 (4.1)	
Region	0 (0)	
Relative income	3316 (15.9)	
Independent bedroom	6853 (32.8)	

a SA: sexual abuse.

b NLE: negative life events.

c IA: internet addiction.

d STB: suicidal thoughts and behaviors.

Following multiple imputation of missing values, the sensitivity analysis yielded results consistent with the primary analysis, demonstrating robustness. The outcomes after imputation are presented in Table S3 in Multimedia Appendix 1.

## Discussion

### Principal Findings

This study revealed that STB were positively associated with SA among adolescents. Notably, NLE and IA independently mediated the path from SA to STB. Furthermore, NLE and IA played a serial mediating role in the relationship between SA and STB. The study constructs a new theoretical model with adolescents as the research object and provides guidance and recommendations to develop interventions for adolescent suicide.

### SA and STB

This study’s findings support hypothesis 1 that SA would be positively associated with STB among adolescents, suggesting that adolescents with a history of SA are at a higher risk of developing STB. Our results align with previous cross-sectional and longitudinal studies that noted a consistent association between childhood SA and an increased risk of suicide attempts [11,40,41]. Several biological and psychological theories elucidate how SA increases the risk of STB. According to neuroimaging investigations, childhood abuse is associated with reduced medial prefrontal cortex volume [42], which reduces an individual’s cognitive functioning; the resultant cognitive deficits can directly increase suicide risk [43,44]. Regarding psychological theories, Finkelhor and Browne [45] identified 2 key trauma dynamics resulting from early SA, namely, stigmatization and feelings of betrayal—commonly experienced by survivors of early SA, especially when the abuser is a familiar member or caregiver [46]. These trauma dynamics catalyze depression, shame, and alienation from others, further eroding one’s sense of self-worth and belonging [47]. These negative effects grow, fostering negative emotions and, after a point, self-harming behaviors and even STB [48].

Notably, gender differences exist in the prevalence of SA and STB among adolescent samples in China currently. Specifically, the probability of STB is higher among girls, while the proportion of experiencing SA is higher among boys [41,49,50]. This pattern likely does not reflect absolute differences in incidence rates but rather profoundly reveals gendered expressions of risk behaviors and sociocultural cognitive biases [51,52]. More concretely, the higher reported rate of suicidal ideation among women may be partly attributable to social norms that more readily permit them to acknowledge and express internalized emotional distress [53]. Conversely, the lower reported rate of SA among male individuals is more likely rooted in the widespread societal neglect of male survivors, the heightened stigma associated with disclosure, and the potential limitations of existing measurement tools in capturing trauma experiences specific to male survivors [50]. It is recommended to focus on the underidentified SA trauma among male adolescents and its potential externalizing behavioral consequences, as well as the clearer pathway from internalizing NLE to suicidal risk among female adolescents.

### Mediating Effect of NLE

This study’s findings verified that NLE mediated the relationship between SA and STB among adolescents, thus supporting hypothesis 2. To our knowledge, previous literature on the mediating role of NLE in the association between SA and STB is scant. Our study expands this field of research and fills a gap in this area. Notably, SA inflicts profound psychological trauma on survivors, which profoundly disrupts their daily lives and interpersonal relationships [54,55]. Furthermore, survivors may grapple with confusion and negative perceptions of their identities and sexual orientations, accompanied by feelings of shame and guilt [56,57]. Moreover, this trauma may impair survivors’ academic, occupational, and daily functioning, as well as precipitate the onset of long-term health issues, including sexually transmitted infections, unintended pregnancies, chronic pain, and physical disabilities [54,58-62].

This study found that NLE were significantly positively correlated with STB, which is consistent with previous research [63-67]. Chronic stress from such events results in depression or despair [68], negative coping strategies, and, ultimately, suicidal thoughts [69]. Rumination may explain how NLE mediate the relationship between SA and STB, suggesting that individuals abused as children, without learning to manage their emotions, may have a reduced sense of control in adulthood [70]. Facing NLE, they tend to repeatedly consider negative outcomes, thus triggering STB.

### Mediating Effect of IA

In this study, IA was identified as a mediator in the association between SA and STB among adolescents, thus corroborating hypothesis 3. Consistent with prior research, survivors of childhood abuse were found to be more prone to IA, with a strong link observed between SA and childhood trauma [71-73]. Per the social compensation hypothesis, the internet offers a suitable platform for self-expression for those who have faced prolonged NLE [74]. Furthermore, SA creates a dangerous environment for the survivor, thus resulting in numerous negative consequences [75]. Hence, they may hesitate to disclose their true selves in face-to-face interactions. Given the anonymous and concealed nature of the internet, these individuals are highly likely to turn to it as a platform for self-expression and release, which may, in turn, elevate their susceptibility to IA [76]. Additionally, IA may result in a variety of drastic physical, psychological, and social relation changes that adolescents can neither control nor avoid [77]. This cycle can trigger intense stress, potentially culminating in a mental health crisis, self-harm, and suicidal behaviors among adolescents. Moreover, the very online environments they frequent—through activities like gaming, socializing, or watching videos—are linked to increased suicide risk [78]. Within these spaces, discussions that normalize suicide or even share methods are not uncommon, potentially providing susceptible youths with both the impetus and the means for self-harm [79].

### Serial Mediating Effect of NLE and IA

This study supported hypothesis 4, as the association between SA and STB was manifested through a serial mediation pathway involving NLE and IA. Adolescents who have experienced SA are more inclined to adopt negative coping styles when dealing with NLE, such as by frequently using the internet to escape reality, which increases their likelihood of receiving undesirable information, experiencing negative moods, and exhibiting STB [27].

A significant positive correlation was observed between NLE and IA—consistent with previous research [80]. First, when faced with NLE, adolescents tend to adopt negative coping styles to alleviate negative emotions; adolescents commonly use the internet for emotional venting [80]. Owing to the lack of social support from family, friends, or other people, adolescents choose to use the internet to avoid the unpleasantness and pain of reality and forget their troubles temporarily [81]. Hence, IA may be a compensatory approach to deal with NLE [82]. Adolescents can seek help on the internet and gain a sense of accomplishment through gaming and entertainment. Adolescents who have experienced NLE are more likely to feel lonely, unaccepted, or have low self-confidence and may, thus, seek a sense of belonging and recognition online [67,83]. Such virtual social relationships may increase their feelings of dependence on the internet [84].

### Suggestions for Future Research

Based on our findings, potential targets for intervention and multipathway mechanisms have been identified. In practice, we propose the following recommendations. First, in clinical assessments, adolescents with a history of SA should undergo routine screening for negative cognitive biases and problematic internet use patterns to facilitate the early identification of high-risk individuals [85]. Second, efforts to enhance adolescent psychological resilience should focus on helping youth modify their cognitive appraisal of NLE and preventing these appraisals from evolving into maladaptive behavioral patterns [86]. Third, modular content addressing IA should be incorporated into adolescent mental health initiatives. This content should aim to help adolescents understand the emotional needs underlying their internet use and collaboratively establish healthy alternative activities and time management plans [87].

### Limitations

This study has several limitations. First, given the cross-sectional design, the mediation model we examined reveals patterns of statistical association among the variables but cannot establish strict causal direction or temporal sequence. Future research should adopt longitudinal or cross-lagged designs to more clearly elucidate the causal timing and dynamic interactions among these variables. Second, there may be reverse causality between the variables or spurious associations due to unmeasured variables. Third, the SA assessment tool used in this study is not a widely validated standardized instrument, and simply summing dichotomous items may not fully capture the complexity of SA experiences. Furthermore, this total score should not be interpreted as a strict interval scale.

### Conclusions

SA is significantly correlated with STB among adolescents, and it indirectly exacerbates suicide risk through the serial mediation of NLE and IA.

This study possesses both theoretical and practical value in the prevention of suicide among adolescents. First, unlike previous research that has largely focused on isolated risk factors or single mediators, this study innovatively constructs and validates a chain mediation model, which reveals the chained mediating roles of NLE and IA between SA and STB. Second, this study systematically elucidates the sequential pathway mechanism from early trauma to psychosocial stress, then to behavioral problems, and ultimately to suicide risk, offering a novel perspective on the complex developmental process from early adversity to extreme outcomes. Finally, the findings provide clear direction and scientific evidence for the development of comprehensive suicide prevention strategies. Future efforts should focus on the prevention of SA and trauma intervention while simultaneously strengthening adolescents’ cognitive reappraisal of negative events and guiding their online behavior.

## Supplementary material

10.2196/85371 Multimedia Appendix 1 Summary of gender differences in participants, multicollinearity test results, and chained mediation imputation results.

10.2196/85371 Checklist 1 JARS checklist.

10.2196/85371 Checklist 2 ASLEC checklist.

## Abbreviations

ASLEC: Adolescent Self-Rating Life Event Checklist
IA: internet addiction
IAT: Internet Addiction Test
JARS: Journal Article Reporting Standards
KMO: Kaiser-Meyer-Olkin
NLE: negative life events
SA: sexual abuse
STB: suicidal thoughts and behaviors
WHO: World Health Organization
