# Supplementary material for: Suicidal Thoughts and Behaviors Among Chinese Adolescents in Relation to Negative Life Events, Internet Addiction, and Sexual Abuse: Cross-Sectional Study
Source: J Med Internet Res. 2026 Mar 25;28:e85371. doi: 10.2196/85371 (PMC13016548; doi:10.2196/85371)
Supplement: Multimedia Appendix 1 [file jmir-v28-e85371-s001.docx]

**Supplementary Material**

**Content**

Table S1. Prevalence and gender differences of sexual abuse and suicidal thoughts and behaviors

Table S2. Multicollinearity Test Results

Table S3. Results of Chained Mediation Imputation

Table S1. Prevalence and gender differences of sexual abuse and suicidal thoughts and behaviors

| Variables | Total | Male  (N= 4838), n(%) | Female  (N= 5826), n(%) | χ^2^ | *P* value |
| --- | --- | --- | --- | --- | --- |
| Sexual abuse |  |  |  | 927.858 | <.001 |
| No | 4,560(42.76%) | 1,294(28.38%) | 3,266(71.62%) |  |  |
| Yes | 6,104(57.24%) | 3,533(58.06%) | 2,560(41.94%) |  |  |
| Suicidal thoughts and behaviors |  |  |  | 40.313 | <.001 |
| No | 8,019(75.20%) | 3,779(47.13%) | 4,240(52.87%) |  |  |
| Yes | 2,645(24.80%) | 1,059(40.04%) | 1,586(59.96%) |  |  |

Table S2. Multicollinearity Test Results

| Variables | SA →NLE | SA+NLE → IA | SA+NLE+IA→STB |
| --- | --- | --- | --- |
|  | VIF | VIF | VIF |
| SA | 1.14 | 1.17 | 1.19 |
| NLE | - | 1.08 | 1.16 |
| IA | - | - | 1.15 |
| STB | - | - | - |
| Age | 1.04 | 1.04 | 1.05 |
| Gender | 1.16 | 1.16 | 1.16 |
| Ethnic group | 1.06 | 1.06 | 1.07 |
| BMI | 1.01 | 1.02 | 1.02 |
| Personality | 1.00 | 1.00 | 1.00 |
| One Child | 1.17 | 1.17 | 1.17 |
| Parent’s marital status | 1.07 | 1.07 | 1.07 |
| Relationship with father | 1.14 | 1.14 | 1.15 |
| Relationship with mother | 1.12 | 1.12 | 1.12 |
| Father’s education level | 1.48 | 1.48 | 1.48 |
| Mother’s education level | 1.54 | 1.54 | 1.54 |
| Region | 1.04 | 1.04 | 1.04 |
| Income status in local | 1.02 | 1.02 | 1.02 |
| Independent bedroom | 1.03 | 1.03 | 1.04 |
| Mean VIF | 1.13 | 1.14 | 1.14 |

Table S3. Results of Chained Mediation Imputation

| Model pathways | β | *P* | Ratio | 95%CI |
| --- | --- | --- | --- | --- |
| Total effect | 0.143 | <.001 |  | (0.127,0.160) |
| Direct effect | 0.100 | <.001 | 69.98% | (0.085,0.116) |
| Total indirect effect | 0.043 | <.001 | 30.02% | (0.038,0.048) |
| Sexual abuse →Negative life events→ Suicidal thoughts and behaviors | 0.019 | <.001 | 13.61% | (0.016,0.023) |
| Sexual abuse → Internet addiction→ Suicidal thoughts and behaviors | 0.018 | <.001 | 12.84% | (0.015,0.022) |
| Sexual abuse →Negative life events→ Internet addiction →Suicidal thoughts and behaviors | 0.005 | <.001 | 3.57% | (0.004,0.006) |
