# Supplementary material for: Suicidal Thoughts and Behaviors Among Chinese Adolescents in Relation to Negative Life Events, Internet Addiction, and Sexual Abuse: Cross-Sectional Study
Source: J Med Internet Res. 2026 Mar 25;28:e85371. doi: 10.2196/85371 (PMC13016548; doi:10.2196/85371)
Supplement: Checklist 2 [file jmir-v28-e85371-s003.docx]

Adolescent Self-Rating Life Event Checklist

| Event | No Occurrence | Mild | Moderate | Severe | Extremely Severe |
| --- | --- | --- | --- | --- | --- |
| 1. Being misunderstood or wrongly accused |  |  |  |  |  |
| 2. Being discriminated against or ignored |  |  |  |  |  |
| 3. Failing an exam or unsatisfactory academic performance |  |  |  |  |  |
| 4. Having conflicts with classmates or friends |  |  |  |  |  |
| 5. Significant changes in daily routines (e.g., diet, rest) |  |  |  |  |  |
| 6. Disliking school |  |  |  |  |  |
| 7. Relationship problems or breakup |  |  |  |  |  |
| 8. Being far away from family and unable to reunite |  |  |  |  |  |
| 9. Heavy study load |  |  |  |  |  |
| 10. Tension with teachers |  |  |  |  |  |
| 11. Suffering from a serious illness |  |  |  |  |  |
| 12. Family or friends suffering from a serious illness |  |  |  |  |  |
| 13. Death of a family member or friend |  |  |  |  |  |
| 14. Being robbed or losing something |  |  |  |  |  |
| 15. Losing face in public |  |  |  |  |  |
| 16. Family financial difficulties |  |  |  |  |  |
| 17. Family conflicts |  |  |  |  |  |
| 18. Failure in expected awards (e.g., "Best Student") |  |  |  |  |  |
| 19. Being criticized or punished |  |  |  |  |  |
| 20. Changing school or taking a leave of absence |  |  |  |  |  |
| 21. Being fined |  |  |  |  |  |
| 22. Pressure of further education or academic advancement |  |  |  |  |  |
| 23. Fighting with others |  |  |  |  |  |
| 24. Being scolded or punished by parents |  |  |  |  |  |
| 25. Parents putting pressure on academic performance |  |  |  |  |  |
| 26. Unexpected shock or accident |  |  |  |  |  |
| 27. Other setbacks or difficulties |  |  |  |  |  |
